# Chemical Composition, Antioxidant, and Antimicrobial Activities of Lichen *Umbilicaria cylindrica* (L.) Delise (Umbilicariaceae)

**DOI:** 10.1155/2012/452431

**Published:** 2011-09-07

**Authors:** Nedeljko T. Manojlovic, Perica J. Vasiljevic, Pavle Z. Maskovic, Marina Juskovic, Gordana Bogdanovic-Dusanovic

**Affiliations:** ^1^Department of Pharmacy, Medical Faculty, University of Kragujevac, 34 000 Kragujevac, Serbia; ^2^Department of Biology and Ecology, Faculty of Sciences and Mathematics, University of Nis, 18 000 Nis, Serbia; ^3^Faculty of Agronomy, University of Kragujevac, 32 000 Čačak, Serbia; ^4^College of Applied Professional Studies, 17000 Vranje, Serbia

## Abstract

The phytochemical analysis of methanol and chloroform extracts of *Umbilicaria cylindrica* was determined by HPLC-UV method. The predominant phenolic compound in both extracts was depsidone, salazinic acid (1). Besides salazinic acid, the tested extracts of *U. cylindrica* contain norstictic acid (2), methyl-**β**-orcinol carboxylate (3), ethyl haematommate (4), atranorin (5), and usnic acid (6), in different amounts and relations. The lichen extracts showed comparable and strong antioxidant activity, exhibited higher DPPH and hydroxyl radical scavengings, chelating activity, and inhibitory activity towards lipid peroxidation. The lichen extracts demonstrated important antimicrobial activity against eight strains with MIC values from 15.62 to 62.50 **μ**g/mL. This is the first report of the detail chemical composition and antioxidant activity of the lichen *Umbilicaria cylindrica*, and the results suggest that this lichen can be used as a new source of the natural antioxidants and the substances with antimicrobial features.

## 1. Introduction

Lichens are valuable plant resources and are used as medicines, food, fodder, dyes perfume, spice, and for miscellaneous purposes. More than one thousand primary and secondary metabolites with identified structures are currently known in lichens [1].The use of lichens in medicine is based on the fact that they contain unique and varied biologically active substances, mainly with antimicrobial actions. These substances are used in lichen chemotaxonomy (i.e., their classification in terms of chemical features), and they are of interest as natural antibiotics. Lichen metabolites exert a wide variety of biological actions including antibiotic, antimycotic, antiviral, anti-inflammatory, analgesic, antipyretic, antiproliferative, and cytotoxic effects [1–6]. Lichens have been found to contain a variety of secondary lichen substances with strong antioxidant activity. These are substances which have high ability to scavenge toxic free radicals due their phenolic groups. Even though these manifold activities of lichen metabolites have now been recognized, their therapeutic potential has not yet been fully explored and thus remains pharmaceutically unexploited. 

Depsides, tridepsides, and tetradepsides consist of two, three, and four hydroxybenzoic acid residues linked by ester groups. They are the most numerous classes of secondary metabolites in lichens. More than one hundred lichen compounds are depsidones, which have an additional ether bond between aromatic rings. Depsidones in lichen are believed to arise by oxidative cyclisation of depsides. It has been found that depsidones are more efficient antioxidants than depsides. Many depsides reported in the literature have been found to possess important physiological properties. Hidalgo et al. [7] reported the antioxidant activity of some depsides, such as atranorin (isolated from *Placopsis *sp.) and divaricatic acid (isolated from *Protousnea malacea*), and depsidones, such as pannarin (isolated from *Psoroma pallidum*) and 1′-chloropannarin (isolated from *Erioderma chilense*). The higher efficiency of the depsidones could be related to a larger incorporation into lipidic microdomains [7]. Depsidone and depside compounds such as pannarin, 10-chloropannarin, and sphaerophorin, tested in cell cultures of lymphocytes, were shown to have a higher cytotoxic effect than colchicine [8]. The depsidones salazinic acid, stictic acid, and psoromic acid were the most apoptotic active derivatives among 15 lichen compounds evaluated on primary cultures of rat hepatocytes. Ranković et al. [9] screened the antimicrobial properties of acetone, methanol, and aqueous extracts of the lichens *Lasallia pustulata*, *Parmelia sulcata*, *Umbilicaria crustulosa*, and *Umbilicaria cylindrica*.

Previous research has shown that some of the Umbilicariaceae, characterized by the production of gyrophoric and lecanoric acids, also contain an additional tridepside (umbilicaric acid), *β*-orcinol depsidones, and anthraquinone pigments [10]. In Serbia, *U. cylindrica *could be found in Babin zub (Mt. Stara planina) on rocks.

The aim of the present work was to identify secondary metabolites of *Umbilicaria cylindrica* by HPLC-UV and to evaluate the antioxidant capacity and antimicrobial activity of the methanol and chloroform extracts from this lichen.

## 2. Material and Methods

### 2.1. Lichen Material

The lichen material was collected from Mt. Stara planina (Babin zub) in Serbia during July 2009. A voucher specimen (HMN 5491) has been deposited at the Herbarium Moesiacum Nis in the Department of Biology and Ecology, Faculty of Sciences and Mathematics, University of Nis, Serbia.

### 2.2. Preparation of the Lichen Extracts

The lichen material was air-dried at room temperature (26°C) for one week, after which it was ground to a uniform powder. Both extracts (methanol and chloroform) were prepared by soaking 500 g dry powdered lichen material in 2000 mL of solvent at room temperature for 3 days. The extracts were filtered through a Whatman no. 42 (125 mm) filter paper and concentrated using a rotary evaporator.

### 2.3. Instrumentation and Conditions

**Figure 1:**
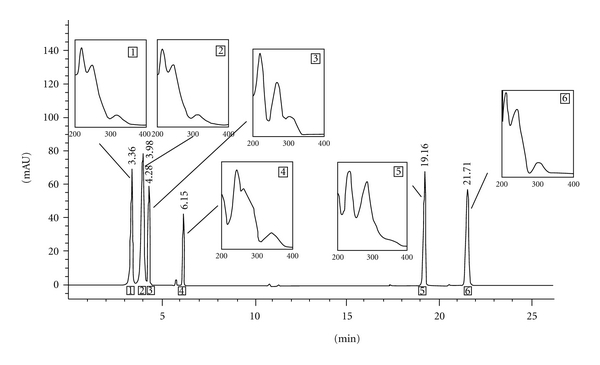
Chromatogram of the standards used for identification of the compounds present in *Umbilicaria cylindrica*. The UV spectra of these compounds (200–400 nm) can be observed in detail.

Reverse phase HPLC analysis was carried out on an Agilent 1200 Series HPLC instrument with C18 column (C18; 25 cm × 4.6 mm, 10 m) and a UV spectrophotometric detector with methanol-water-phosphoric acid (80 : 20 : 0.9, v/v/v) solvent. Methanol was of HPLC grade and was purchased from Merck (Darmstadt, Germany). Phosphoric acid was analytical-grade reagent. Deionized water used throughout the experiments was generated by a Milli-Q academic water purification system (Milford, MA, USA). The sample injection volume was 10 *μ*L. The flow rate was 1.0 mL/min. The standards used were obtained from the following sources: salazinic acid ((1): *t*_R_ = 3.36 ± 0.10 min) was isolated from *Lobaria pulmonaria*, norstictic acid ((2): *t*_R_ = 3.98 ± 0.20 min) isolated from lichen *Ramalina farinacea*, ethyl haematommate ((3): *t*_R_ = 4.28 ± 0.20 min) and methyl-*β*-orcinol carboxylate (evernyl or methyl atrarate), ((4): *t*_R_ = 6.15 ± 0.30 min), isolated from *Pseudevernia furfuracea*, usnic acid ((5): *t*_R_ = 19.16 ± 0.30 min), and atranorin ((6): *t*_R_ = 21.71 ± 0.30 min) from lichen *Evernia prunastri*. The retention times and UV spectra of these standards are shown in Table 2 and Figure 1.

### 2.4. Determination of the Total Phenolics

The total phenolics content was determined using the Folin-Ciocalteau method [11]. Extract was diluted to the concentration of 1 mg/mL, and aliquots of 0.5 mL were mixed with 2.5 mL of FC reagent (previously diluted 10-fold with distilled water) and 2 mL of NaHCO_3_ (7.5%). After 15 min of staying at the 45°C the absorbance was measured at 765 nm on spectrophotometer versus blank sample. Total phenols were determined as gallic acid equivalents (mg GA/g extract), and the values are presented as means of triplicate analyses.

### 2.5. Determination of Total Antioxidant Capacity

The total antioxidant activity of the *Umbilicaria cylindrica* extract was evaluated by the phosphomolybdenum method [12]. The assay is based on the reduction of Mo (VI)-Mo (V) by the antioxidant compounds and subsequent formation of a green phosphate/Mo (V) complex at acid pH. 0.3 mL of sample extract was combined with 3 mL of reagent solution (0.6 M sulfuric acid, 28 mM sodium phosphate and 4 mM ammonium molybdate). The tubes containing the reaction solution were incubated at 95°C for 90 min. Then the absorbance of the solution was measured at 695 nm using spectrophotometer against blank after cooling to room temperature. Methanol (0.3 mL) in the place of extract was used as the blank. Ascorbic acid (AA) was used as standard and the total antioxidant capacity is expressed as milligrams of ascorbic acid per gram of the dry extract.

### 2.6. Determination of DPPH Free Radical Scavenging Activity

The method used by the authors of [13] was adopted with suitable modifications from [14]. DPPH (8 mg) was dissolved in MeOH (100 mL) to obtain a concentration of 80 *μ*g/mL. Serial dilutions were carried out with the stock solution (1 mg/mL) of the extract. Solutions (2 mL each) were then mixed with DPPH (2 mL) and allowed to stand for 30 min for any reaction to occur, and the absorbance was measured at 517 nm. Ascorbic acid (AA), gallic acid (GA), and butylated hydroxytoluene (BHT) were used as reference standards and dissolved in methanol to make the stock solution with the same concentration (1 mg/mL). Control sample was prepared containing the same volume without test compounds or reference antioxidants. 95% methanol was used as blank. The DPPH free radical scavenging activity (%) was calculated using the following equation:

(1) $$\%\;\text{inhibition}=\frac{\text{Ac}-\text{As}}{\text{Ac}}\times 100.$$

The IC_50_ value, which is the concentration of the test material that reduces 50% of the free radical concentration, was calculated as *μ*g/mL through sigmoidal dose-response curve.

### 2.7. Determination of the Inhibitory Activity toward Lipid Peroxidation

The antioxidant activity was determined by the thiocyanate method [15]. Serial dilutions were carried out with the stock solution (1 mg/mL) of the extracts, and 0.5 mL of each solution was added to linoleic acid emulsion (2.5 mL, 40 mM, pH 7.0). The linoleic acid emulsion was prepared by mixing 0.2804 g linoleic acid, 0.2804 g Tween-20 as emulsifier in 50 mL 40 mM phosphate buffer and the mixture was then homogenized. The final volume was adjusted to 5 mL with 40 mM phosphate buffer, pH 7.0. After incubation at 37°C in the dark for 72 h, a 0.1 mL aliquot of the reaction solution was mixed with 4.7 mL of ethanol (75%), 0.1 mL FeCl_2_ (20 mM), and 0.1 mL ammonium thiocyanate (30%). The absorbance of this mixture was measured at 500 nm, after it was stirred for 3 min. Ascorbic acid, gallic acid, *α*-tocopherol, and BHT were used as a reference compounds. To eliminate the solvent effect, the control sample, which contained the same amount of solvent added to the linoleic acid emulsion in the test sample and reference compound, was used. Inhibition percent of linoleic acid peroxidation was calculated using the following formula:

(2) $$\%\;\text{inhibition}=\frac{\text{Ac}-\text{As}}{\text{Ac}}\times 100.$$

### 2.8. Measurement of Ferrous Ion Chelating Ability

The ferrous ion chelating ability was measured by the decrease in absorbance at 562 nm of the iron (II)-ferrozine complex [16, 17]. One milliliter of 0.125 mM FeSO_4_ was added to 1.0 mL sample (with different dilutions), followed by 1.0 mL of 0.3125 mM ferrozine. The mixture was allowed to equilibrate for 10 min before measuring the absorbance. The ability of the sample to chelate ferrous ion was calculated relative to the control (consisting of iron and ferrozine only) using the formula:

(3) $$\text{Chelating}\;\text{effect}\;\left(\%\right)=\frac{\text{Ac}-\text{As}}{\text{Ac}}\times 100.$$

### 2.9. Determination of Hydroxyl Radical Scavenging Activity

The ability of* Umbilicaria cylindrica* to inhibit nonsite-specific hydroxyl radical-mediated peroxidation was carried out according to the method described in [18]. The reaction mixture contained 100 *μ*L of extract dissolved in water, 500 *μ*L of 5.6 mM 2-deoxy-D-ribose in KH_2_PO_4_-NaOH buffer (50 mM, pH 7.4), 200 *μ*L of premixed 100 *μ*M FeCl_3_ and 104 mM EDTA (1 : 1 v/v) solution, 100 *μ*L of 1.0 mM H_2_O_2_, and 100 *μ*L of 1.0 mM aqueous ascorbic acid. Tubes were vortexed and incubated at 50°C for 30 min. Thereafter, 1 mL of 2.8% TCA and 1 mL of 1.0% TBA were added to each tube. The samples were vortexed and heated in a water bath at 50°C for 30 min. The extent of oxidation of 2-deoxyribose was estimated from the absorbance of the solution at 532 nm. The percentage inhibition values were calculated from the absorbance of the control (Ac) and of the sample (As), where the controls contained all the reaction reagents except the extract or positive control substance. The values are presented as the means of triplicate analyses.

### 2.10. Statistical Analysis

All the results are presented as mean ± standard deviations of three determinations. Statistical analyses were performed using Student's *t*-test and one-way analysis of variance. Multiple comparisons of means were done by LSD (least significant difference) test. A probability value of 0.05 was considered significant. All computations were made by employing the statistical software (SPSS, version 11.0). IC_50_ values were calculated determined by nonlinear regression analysis from the sigmoidal dose-response inhibition curve. 

### 2.11. Antimicrobial Activity

#### 2.11.1. Chemicals

All chemicals and reagents were of analytical grade and were purchased from Sigma Chemical Co. (St. Louis, MQ, USA), Aldrich Chemical Co. (Steinheim, Germany), and Alfa Aesar (Karlsruhe, Germany).

#### 2.11.2. Spectrophotometric Measurements

Spectrophotometric measurements were performed using a UV-VIS spectrophotometer MA9523-SPEKOL 211 (ISKRA, Horjul, Slovenia).

#### 2.11.3. Test Microorganisms

The antimicrobial activity of the plant extract was tested *in vitro* against the following bacteria:* Staphylococcus aureus* ATCC 25923, *Klebsiella pneumoniae* ATCC 13883, *Escherichia coli* ATCC 25922, *Proteus vulgaris* ATCC 13315, *Proteus mirabilis* ATCC 14153, and *Bacillus subtilis* ATCC 6633, and fungi: *Candida albicans* ATCC 10231 and *Aspergillus niger* ATCC 16404. The fungi were reseeded on potato-glucose agar, on which they developed for 7 days at room temperature of 20°C under alternating day/night light conditions. They were reseeded on a new potato-glucose substrate, on which they developed for another 7 days. The reseeding procedure was performed four times, after which the pure cultures needed for determination were obtained. The identification of the test microorganisms was confirmed by the Laboratory of Mycology, Department of Microbiology, Institute Torlak, Belgrade, Serbia.

#### 2.11.4. Minimum Inhibitory Concentration (MIC)

The minimal inhibitory concentrations (MIC) of the extract and cirsimarin against tested bacteria were determined based on a microdilution method in 96 multiwell microtiter plates [19]. All tests were performed in Muller-Hinton broth (MHB) with the exception of the yeast when Sabouraud dextrose broth was used. A volume of 100 *μ*L stock solutions of oil (in methanol, 200 *μ*L/mL) and cirsimarin (in 10% DMSO, 2 mg/mL) was pipette into the first row of the plate. To all other wells 50 *μ*L of Mueller Hinton or Sabouraud dextrose broth (supplemented with Tween 80 at a final concentration of 0.5% (v/v) for analysis of oil) was added. A volume of 50 *μ*L from 1st test wells was pipetted into the 2nd well of each microtiter line, and then 50 *μ*L of scalar dilution was transferred from the 2nd to the 12th well. To each well 10 *μ*L of resazurin indicator solution (prepared by dissolving a 270 mg tablet in 40 mL of sterile distilled water) and 30 *μ*L of nutrient broth were added. Finally, 10 *μ*L of bacterial suspension (10^6^ CFU/mL) and yeast spore suspension (3 × 10^4^ CFU/mL) was added to each well. For each strain, the growth conditions and the sterility of the medium were checked. Standard antibiotic amracin was used to control the sensitivity of the tested bacteria whereas ketoconazole was used as control against the tested yeast. Plates were wrapped loosely with cling film to ensure that bacteria did not become dehydrated and prepared in triplicate, and then they were placed in an incubator at 37°C for 24 h for the bacteria and at 28°C for 48 h for the yeast. Color change was then assessed visually. Any color change from purple to pink or colorless was recorded as positive. The lowest concentration at which color change occurred was taken as the MIC value. The average of 3 values was calculated and that was the MIC for the tested compounds and standard drug.

## 3. Results and Discussion

**Figure 2:**
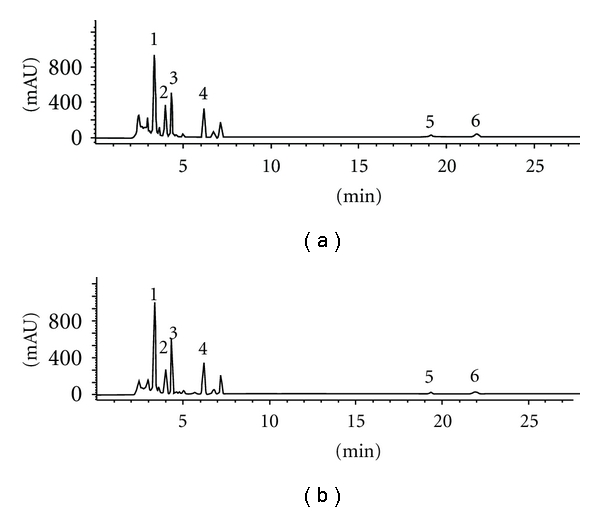
HPLC chromatograms acquired at 254 nm of the methanol (a) and chloroform (b) extracts of *Umbilicaria cylindrica*. Chromatographic peaks identities are reported in Table 2.

 Chromatograms for standards and *Umbilicaria cylindrica* methanol and chloroform extracts eluted by HPLC are represented in Figures 1 and 2. As it is evidenced in the chromatograms, there were the presence of depsidones as the most abundant substance class in the extracts examined. As the most abundant depsidone, salazinic acid (SAL; *t*_R_ = 3.36 ± 0.10 min) was identified. This acid has previously been reported as a constituent of some *Usnea* species [6, 20]. 

**Scheme 1:**
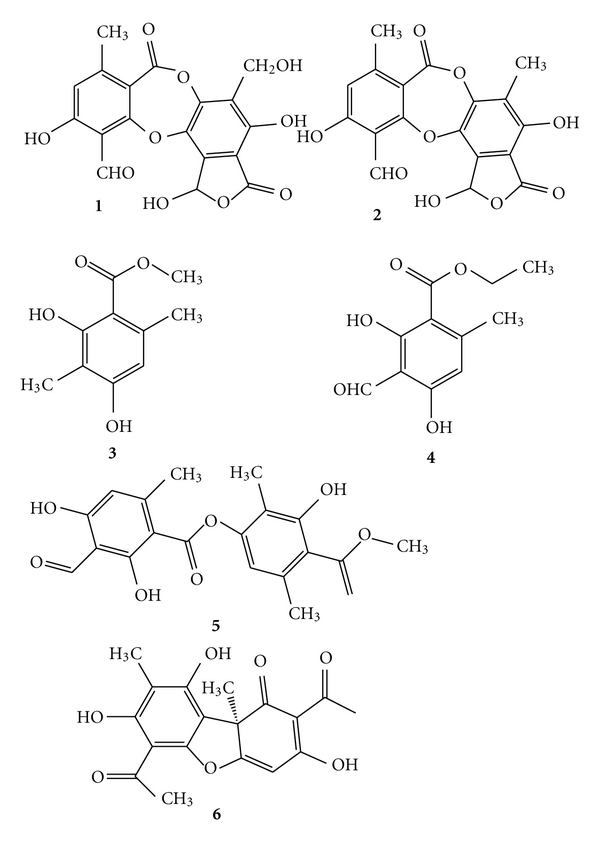
Structures of the identified compounds.

Comparing the *t*_R_ values and the UV spectra from HPLC-UV (Scheme 1) with those of authentic substances, it is readily confirmed that the major metabolites of the lichen (besides salazinic acid) are norstictic acid ((2): *t*_R_ = 3.98 ± 0.20 min), methyl-*β*-orcinol carboxylate ((3): *t*_R_ = 4.28 ± 0.20 min), ethyl haematommate ((4): *t*_R_ = 6.15 ± 0.30 min), usnic acid ((5): *t*_R_ = 19.16 ± 0.30 min), and atranorin ((6): *t*_R_ = 21.71 ± 0.30 min). Compounds **1** and **2** belonging to the depsidones while **3** and **4** are monocyclic aromatic compounds. Atranorin is depside and usnic acid is antibiotic with dibenzofuran structure. The UV spectra of depsidones have 3 absorption maxima and are dissimilar from those of depsides and monocyclic compounds. The UV spectra of salazinic acid are very similar to those of norstictic acid. Absorbance maxima at 234 and 282 nm are characteristic for usnic acid. Except salazinic acid, other compounds were found in the extracts in small amounts. 

Identification of these compounds was achieved by comparison of their *t*_R_ values with the standard substances previously isolated from lichens. The UV absorbance spectral data (200–400 nm) also corresponded with those of standards and those found in [21].  Table 1 shows the retention time of the detected lichen substances and their absorbance maxima (nm). All compounds are identified for the first time in this lichen. 

Phenolic compounds have been reported to be associated with antioxidative action in biological systems, mainly due to their red-ox properties, which can play an important role in absorbing and neutralizing free radicals, quenching singlet and triplet oxygen, or decomposing peroxides [22]. The results of determination of total phenolic and antioxidant capacity are given in Table 2. Total phenolic contents were determined and amounted to 79.2 ± 0.59 mg GA/g and 71.32 ± 0.87 mg GA/g, for methanol and chloroform extracts, respectively. The results showed that the methanolic and chloroform extracts possess antioxidant activity, with total antioxidant capacity of 74.65 ± 0.75 *μ*g AA/g and 68.35 ± 0.15 *μ*g AA/g, respectively. 

The assessment of DPPH scavenging activity showed that both tested extracts were able to scavenge this radical (Table 3). The chloroform extract displayed a higher activity (31.34 ± 1.10 *μ*g/mL) than methanol extract 34.45 ± 1.15 *μ*g/mL). Although this scavenging effect was lower than that of BHT (Table 3), it was stronger than the antioxidant activity reported in many other lichen species [23]. 

Results demonstrated also that all tested extracts exhibited significant inhibitory activity towards lipid peroxidation (from 29.31 ± 0.65 to 35.36 ± 1.68 *μ*g/mL). The results of metal chelating activity are also shown in the Table 4 and these values were very similar for both tested extracts.

The results of determination of hydroxyl radical scavenging activity (Table 4) showed that IC_50_ values were 83.34 ± 0.35 *μ*g/mL and 89.11 ± 0.29 *μ*g/mL *μ*g/mL for methanolic and chloroform extracts, respectively. These results revealed that the methanol and chloroform extracts of *U. cylindrica* organs were free radical scavengers, acting possibly as primary antioxidants. The strong antioxidant activity of *U. cylindrica* assessed by the different systems could be attributed to their high total polyphenolic contents, since a positive correlation between phenolic composition and antioxidant activity was proved [24]. Thus, antioxidant property of the lichen could be attributed to the significant amount of depsidones, especially salazinic acid. Other minor phenolic compounds should not be neglected, since synergy of the different chemicals with each other should be taken into consideration for the biological activity. The presence of the phenolic groups in the lichen metabolites is considered to be a key element for the antioxidative efficiency [25]. Salazinic acid possesses four hydroxyl groups (two phenolic groups) in the molecule which probably play an important role in expression of their antioxidant activity. The results of the antimicrobial activity obtained by the dilution method are reported in Table 4. Minimum inhibitory concentrations were determined for eight selected indicator strains. The results showed a relatively strong inhibitory activity, depending on the sort of lichens, related to the tested bacteria and fungi. The antimicrobial activity of extracts of *Umbilicaria cylindrica* was within the concentration range from 16.62 *μ*g/mL to 62.50 *μ*g/mL. The antimicrobial activity of the tested extracts was better than the activity of the protolichesteric acid-containing extracts of *Cetraria aculeata* [26] and many other lichen extracts [5, 23, 27, 28]. The relevant antimicrobial activity of *U. cylindrica *probably depends on the presence of two depsidones, methyl-*β*-orcinol carboxylate and ethyl haematommate, but a small amount of usnic acid, as an important antibiotic, and atranorin in the extracts was also confirmed. In conclusion, this is the first study focused on the detail chemical composition and antioxidant activity of* U. cylindrica.*

The methanol and chloroform extracts of the lichen showed significant antioxidant and antimicrobial activity in different assays *in vitro*. Two monocyclic aromatic compounds, two depsidones, one depside, and usnic acid were identified, and salazinic acid was the dominant phenolic compound in the lichen. Although these compounds have already been reported for some other lichen species, this is the first report for* U. cylindrica*. The present study provides data for supporting the use of *U. cylindrica * extracts as natural antimicrobial and antioxidant agents and confirms that these extracts represent a significant source of phenolic compounds. Future investigation will be focused on isolation of phenolic compounds and determination of their biological activities *in vitro* and *in vivo*. Since this lichen has a bigger content of the phenol components which were proven to have numerous biological effects including the antioxidant activity, they can be of a big importance in the food industry, given the fact that they keep the oxidative processes, that way improving the quality and its nutritional value, so they can be used as additives.

## Figures and Tables

**Table 1:** Retention time of the examined lichen substances and their absorbance maxima (nm).

Peaks no.	Compound	Retention time (*t*_R_ ± SD)* (min)	Absorbance maxima (nm) UV spectrum
1	Salazinic acid	3.36 ± 0.01	213, 238, 312
2	Norstictic acid	3.98 ± 0.20	212, 238, 310
3	Methyl-*β*-orcinol carboxylate	4.28 ± 0.05	218, 267, 303
4	Ethyl haematommate	6.15 ± 0.20	240, 258, 285^m^, 337
5	Usnic acid	19.16 ± 0.30	234, 282
6	Atranorin	21.71 ± 0.30	210, 252, 321^m^

*Values are the means of three determinations ± SD, m: minor absorbance maximum.

**Table 2:** Total phenolic and total antioxidant capacity of the examined extracts.

	Total phenolics (mg GA/g)	Total antioxidant capacity (*μ*g AA/g)
Methanol extract	79.2 ± 0.59	74.65 ± 0.75
Chloroform extract	71.32 ± 0.87	68.35 ± 0.15

**Table 3:** The antioxidant activity of the examined extracts.

Plant species	^ a^IC_50_ (*μ*g/mL)			
	DPPH scavenging activity	Inhibitory activity against lipid peroxidation	Metal chelating activity	Hydroxyl radical scavenging activity
*Methanol extract*	34.45 ± 1.15	35.36 ± 1.68	45.91 ± 0.88	89.11 ± 0.29
*Chloroform extract*	31.34 ± 1.10	29.31 ± 0.65	39.46 ± 0.78	83.34 ± 0.35
Gallic acid	3.79 ± 0.69	255.43 ± 11.68	—	59.14 ± 1.10
Ascorbic acid	6.05 ± 0.34	>1000	—	160.55 ± 2.31
BHT	15.61 ± 1.26	1.00 ± 0.23	—	33.92 ± 0.79
*α*-Tocopherol	—	0.48 ± 0.05	—	—

^a^IC_50_ values were determined by nonlinear regression analysis. Results are mean values ± SD from three experiments.

**Table 4:** Minimum inhibitory concentrations (MIC) of the methanol and ethylacetate extracts of *Umbilicaria cylindrica*.

MIC *μ*g/mL				
*Bacterial and fungal strains*	Methanol extract	Ethylacetate extract	Amracin	Ketoconazole
*Staphylococcus aureus ATCC 25923*	15.62	31.25	0.97	—
*Klebsiella pneumoniae ATCC 13883*	31.25	31.25	0.49	—
*Escherichia coli ATCC 25922*	31.25	15.62	0.97	—
*Proteus vulgaris ATCC 13315*	62.50	62.50	0.49	—
*Proteus mirabilis ATCC 14153*	62.50	31.25	0.49	—
*Bacillus subtilis ATCC 6633*	15.62	31.25	0.24	—
*Candida albicans ATCC 10231*	31.25	31.25	—	1.95
*Aspergillus niger ATCC 16404*	15.62	31.25	—	0.97
